# Growing CeO_2_ Nanoparticles Within the Nano-Porous Architecture of the SiO_2_ Aerogel

**DOI:** 10.3389/fchem.2020.00057

**Published:** 2020-02-07

**Authors:** Francesco Caddeo, Danilo Loche, Maria F. Casula, Anna Corrias

**Affiliations:** ^1^School of Physical Sciences, University of Kent, Canterbury, United Kingdom; ^2^Department of Mechanical, Chemical and Material Engineering, University of Cagliari, Cagliari, Italy

**Keywords:** ceria, aerogel, nanoporous materials, nanocomposites, surfactants

## Abstract

In this study, new CeO_2_-SiO_2_ aerogel nanocomposites obtained by controlled growth of CeO_2_ nanoparticles within the highly porous matrix of a SiO_2_ aerogel are presented. The nanocomposites have been synthesized via a sol-gel route, employing cerium (III) nitrate as the CeO_2_ precursor and selected surfactants to control the growth of the CeO_2_ nanoparticles, which occurs during the supercritical drying of the aerogels. Samples with different loading of the CeO_2_ dispersed phase, ranging from 5 to 15%, were obtained. The nanocomposites showed the morphological features typical of the SiO_2_ aerogels such as open mesoporosity with surface area values up to 430 m^2^·g^−1^. TEM and XRD characterizations show that nanocrystals of the dispersed CeO_2_ nanophase grow within the aerogel already during the supercritical drying process, with particle sizes in the range of 3 to 5 nm. TEM in particular shows that the CeO_2_ nanoparticles are well-distributed within the aerogel matrix. We also demonstrate the stability of the nanocomposites under high temperature conditions, performing thermal treatments in air at 450 and 900°C. Interestingly, the CeO_2_ nanoparticles undergo a very limited crystal growth, with sizes up to only 7 nm in the case of the sample subjected to a 900°C treatment.

## Introduction

Silica aerogels are extremely low density and highly porous materials that feature a 3-dimensional randomly organized network of silica nanoparticles filled with air, constituting up to 98% of the total weight (Gesser and Goswami, 1989; Dorcheh and Abbasi, 2008). These materials are prepared via sol-gel synthesis and subsequent drying of the gel in special conditions that avoid the collapse of their fine porous structure. Their open porous architecture comprises mainly mesopores and macropores, with an average size of 20 nm and a surface area that ranges from 300 to 1,000 m^2^·g^−1^. Due to these microstructural features, silica aerogels are well-known for a number of extraordinary properties such as high optical transmission, high open porosity, low dielectric constant, low refractive index, and extremely low thermal conductivity (Malfait et al., 2015; Wan et al., 2018). These properties enable the exploitation of silica aerogels in many technological areas, such as air and water purification (Cao et al., 2006; Loche et al., 2016), thermal insulation in buildings and space vehicles (Fesmire, 2006; Lamy-Mendes et al., 2018), sensing and catalysis (Amonette and Matyáš, 2017).

The open nanoporous architecture and high surface area of silica aerogels, combined with their high thermal stability and the possibility to be synthesized as monoliths, ensure that silica aerogels have a high potential as a support for heterogeneous catalysts for gas-phase reactions. In this field, nanocomposite aerogels featuring a transition metal or metal-oxide nanophase dispersed within the backbone of the porous silica matrix are proposed, taking advantage of the properties of both the porous aerogel and the dispersed nanophase (Amonette and Matyáš, 2017). Relevant examples include the preparation of Ag-SiO_2_ aerogel nanocomposites for selective oxidation of benzene to phenol (Ameen et al., 2007); Fisher-Tropsch synthesis over Co-SiO_2_ aerogel catalysts (Dunn et al., 2005); and TiO_2_-SiO_2_ aerogels for photocatalytic air purification (Yoda et al., 2000).

Most of the methods found in the literature for the synthesis of these aerogel nanocomposites have several drawbacks, including long gelation times and poor control over the loading and dispersion of the supported nanophase. In this regard, our group developed a 2-step acid-base catalyzed co-gelation method that allows one to obtain aerogels with short gelation times and extensive control over the loading and homogeneity of the dispersed nanophase (Casula et al., 2007). The flexibility of this method allowed the preparation of silica aerogels with a variety of dispersed phases, including metal alloys (Casu et al., 2008; Marras et al., 2015), metal-oxides (Casu et al., 2007; Carta et al., 2009) and graphene (Loche et al., 2016). These nanocomposites showed extraordinary stabilities during harsh thermal treatments and catalytic processes (Vanyorek et al., 2011; Loche et al., 2012; Marras et al., 2016). In particular, the crystalline dispersed nanoparticles, that are held apart by the silica porous matrix, do not tend to sinter, which is one of the main issues in catalysis when using unsupported nanoparticles.

Cerium dioxide (CeO_2_, ceria) is an irreplaceable component in the automotive three-way catalysts and currently is widely studied for many industrially relevant catalytic processes, such as the CO oxidation (Suchorski et al., 2008; Yang et al., 2011), the water gas shift reaction (Bruix et al., 2012; Carta et al., 2017) and hydrogenation of alkenes (Vilé et al., 2012; Montini et al., 2016). Recently, ceria has been also proposed as suitable catalysts or catalytic support for the conversion of CO_2_ (Wang et al., 2016; Boaro et al., 2019). The catalytic activity of ceria is strongly linked to its ability to form a high number of surface oxygen vacancies and Ce^3+^ ions that form surface frustrated Lewis pairs (Zhang et al., 2017) and that correlate with high values of oxygen storage capacity (Li et al., 2016). These properties are especially enhanced in the case of ceria nanoparticles and are size and shape dependent (Mai et al., 2005; Zhang et al., 2011). Moreover, it has been demonstrated that the concentration of oxygen vacancies in ceria nanoparticles with an average size of 4 nm is two orders of magnitude higher than in 60 nm nanoparticles (Zhou and Huebner, 2001).

One of the main problems of metal-oxides during catalytic processes is their tendency to sinter with the consequent loss of their surface area. In the case of ceria, this effect is particularly detrimental due to the consequent loss of its ability to form a high concentration of oxygen vacancies. The growth of CeO_2_ nanoparticles within the nanoporous architecture of a silica aerogel might be a valid solution to this problem while at the same time taking advantage of the aforementioned textural properties of the silica aerogel, which are highly desired in gas-phase heterogeneous catalysis. To the best of our knowledge, CeO_2_-SiO_2_ aerogel composites have been previously reported only in two studies (Dutta et al., 2006; Posada et al., 2019). However, in these works, the dispersion of the ceria phase was obtained through impregnation via solvent exchange, which is a time consuming procedure that does not allow tuning the loading of the dispersed ceria. Moreover, no information was given on the size and distribution of the ceria nanocrystals.

In this paper, we present the synthesis and characterization of new CeO_2_-SiO_2_ aerogels. These nanocomposites were synthesized with the aforementioned 2-steps acid-base catalyzed co-gelation protocol that yields aerogels with an extensive control over the loading and dispersion of the ceria nanoparticles. The use of dodecanoic acid (or hexanoic acid) as surfactant was needed during the synthesis to ensure a homogeneous dispersion of the ceria nanoparticles, and to control their growth. We demonstrate that these new CeO_2_-SiO_2_ aerogel composites show exemplary thermal stability, even when submitted to thermal treatments in air at temperatures as high as 900°C. These conditions were chosen since they are much harsher than those used in industrially relevant catalytic processes, ensuring high stability of these nanocomposites during catalytic reactions. The crystallization of the dispersed ceria nanophase is influenced by the presence of the surfactants and by the textural features of the porous silica, which in turn prevents the sintering of the ceria nanoparticles.

## Experimental

### Samples Preparation

For the preparation of the CeO_2_-SiO_2_ nanocomposites, a synthetic procedure adapted from a protocol previously developed by our group has been used (Casula et al., 2007). This procedure consists of a 2-steps acid-base catalyzed sol-gel synthesis that makes use of a silicon alkoxide—tetraethoxysilane [Si(OC_2_H_5_)_4_, Aldrich 98%, TEOS]—as precursor for the SiO_2_ phase. Under acid-base catalysis, the TEOS undergoes hydrolysis and condensation reactions, leading to a colloidal suspension of SiO_2_ nanoparticles. Appropriate amounts of Cerium (III) nitrate hexahydrate [Ce(NO_3_)_3_ 6H_2_O, Aldrich ≥ 99%] were used as precursor for the CeO_2_. The addition of either dodecanoic acid (C_12_H_24_O_2_, Aldrich 98%) or hexanoic acid (C_6_H_12_O_2_, Aldrich ≥ 98%) as surfactants have been employed to achieve a good dispersion of the nanophase. Nitric acid (HNO_3_, Fisher Chemical, 70%) was used to prepare the hydrolyzing solution needed during the first step of the sol-gel synthesis and urea (CH_4_N_2_O, Aldrich, 99.0–100.5%) was used during the second step as a base to promote the condensation reactions of the TEOS. Absolute ethanol (C_2_H_6_O, Fluka) and distilled water were used as solvents. Under the conditions used, a sol to gel transition occurs after addition of urea, yielding a silica alcogel filled with cerium nitrate solution. The aerogel samples were obtained by high temperature supercritical drying, while a xerogel sample has been obtained by slow evaporation, for comparison.

During a typical synthesis, a solution containing 7.9 ml of TEOS (0.0330 mol) and 3 ml of absolute ethanol was hydrolyzed by adding dropwise 3.965 ml of an acidic stock solution—prepared by mixing 2 ml of nitric acid, 80 ml of absolute ethanol and 130 ml of distilled water—and heating at 50°C for 30 min under reflux. After cooling at room temperature, an ethanolic solution of the appropriate amount of cerium (III) nitrate (calculated to obtain nanocomposites with a loaded dispersed phase of 5, 10, and 15%) was added to the mixture and the resulting solution was stirred for 10 min. Either dodecanoic acid or hexanoic acid were also added to the mixture as surfactants, using Ce^3+^/surfactant molar ratios of 1:1, 1:4 or 1:8 in the case of dodecanoic acid and a molar ratio of 1:4 in the case of hexanoic acid. During the second step, a solution prepared using 3.5130 g of urea (0.0579 mol), 4.92 ml of distilled water and 9 ml of absolute ethanol was added to the pre-hydrolyzed sol and stirred for 10 min. The mixture was then heated at 85°C until a viscous sol was obtained. The sol was poured into a closed glass vial and kept at 40°C for about 24 h to complete the gelation process. The alcogels were submitted a high temperature supercritical drying process in a 300 ml Parr autoclave filled with 70 ml of absolute ethanol. The autoclave was flushed with N_2_ gas at room temperature and then heated at a rate of 5°C min^−1^ up to 250°C and then at 1°C min^−1^ up to 330°C. Once a temperature of 330°C and a pressure of 70 atm were reached, the autoclave was slowly vented to atmospheric pressure. Note that organic solvents like ethanol form explosive mixtures with air in the range of temperatures and pressures used during the supercritical drying. The autoclave needs to be thoroughly flushed with an inert gas (N_2_) before the process, to make sure that all the oxygen has been purged outside the autoclave. Moreover, dangerous gases might be produced during the venting procedure, which needs to be performed under a fume cupboard. Monolithic and highly porous aerogels were obtained. The aerogels and xerogel samples were then thermally treated in air for 1 h at 450 and 900°C. In the following, the samples name will indicate the loading of the ceria and the thermal treatment—i.e., A_15_CeO_2__900 designate an aerogel sample with a 15% loading of ceria thermally treated at 900°C for 1 h. X will be used for the xerogel samples. NT will be used for the sample not thermally treated.

### Characterization

Thermo-gravimetric analysis (TGA) and differential scanning calorimetry (DSC) measurements have been carried out on a NETZSCH STA 409 PC, which allows the simultaneous acquisition of the TGA and DSC curves. Data were collected in the range 25–800°C with a heating rate of 10°C min^−1^, under air flow. Powder X-ray diffraction (XRD) patterns were recorded with a PANalytical Empyrean, with a θ-2θ geometry. The diffractometer is equipped with an X-ray tube with Cu as anode, a focusing germanium monochromator on the incident beam producing a pure Kα1 Cu radiation (λ = 1.5406 Å) and a X'Celerator linear detector. The crystallites average size was calculated using the Scherrer equation; instrumental broadening was determined on a standard LaB_6_ sample. The fitting was performed using the Panalytical HighScore Plus software. The presence of lattice strains was considered negligible. The N_2_-physisorption measurements have been carried out using a Thermo Scientific Surface gas adsorption porosimeter. The samples were degassed under vacuum while thermally treated with a heating rate of 1°C/min up to 200°C for 10 h. The specific surface area (S), the total pore volume (V_P_) and the pore size distributions were assessed by the Branauer-Emmett-Teller (BET) and the Barrett-Joyner-Halenda (BJH) methods, respectively (Brunauer et al., 1938; Barrett et al., 1951). The TEM images were acquired using a Hitachi H-7000 microscope, equipped with a W filament as source of electrons, operating at 125 kV, corresponding to a wavelength of 3.28 10^−2^ Å. The samples were deposited as dry powders on a carbon-coated copper grid. Size distribution was determined using both bright and dark field images and sampling at least 100 particles per sample.

## Results and Discussion

**Figure 1:**
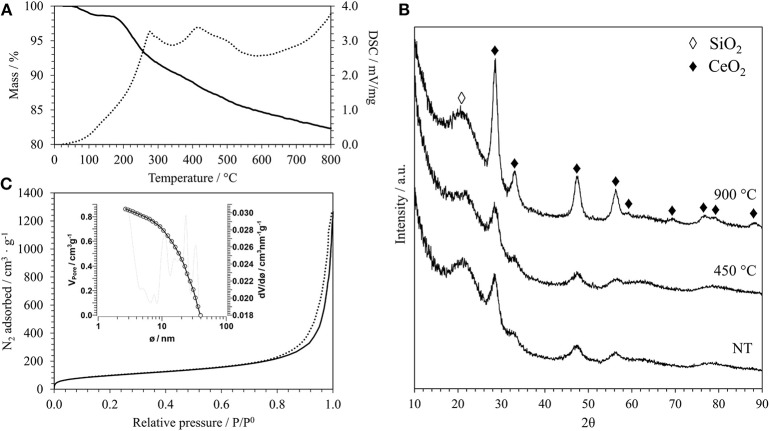
**(A)** TGA/DSC curves of the aerogel sample A_15_CeO_2__NT: TG (bold line), DSC (dashed line). **(B)** XRD patterns of the same sample after supercritical drying (NT) and after thermal treatments at 450°C and 900°C. **(C)** N_2_-physisorption isotherm of the same sample after thermal treatment at 900°C (adsorption branch: bold line; desorption branch: dashed line) and pore size distribution calculated from the desorption branch (inset).

In Figure 1A, as an example of the typical result of the thermal analysis of an aerogel sample after the supercritical drying, the TGA/DSC curves of the aerogel sample with a 15% weight load of CeO_2_ using a Ce^3+^/dodecanoic acid molar ratio of 1:4 are reported. All the aerogels studied in this work show similar TGA/DSC curves, in agreement with previous studies (Loche et al., 2010), even if in this case a surfactant was used. The TG curve shows that the weight loss up to 150°C, which is mainly due to humidity absorbed on the surface of the silica, is very limited (about 3%); this shows that the supercritical drying was successful in removing all the solvent form the pores. The TG curve shows a further weight loss of about 13% up to 800°C. The DSC curve shows two main exothermic peaks, at 227 and 416°C, that are likely due to the combustion of organics since the surface of the silica matrix is partially esterified during the supercritical drying. Organic residues from the incomplete decomposition of the surfactant used during the synthesis might be also present. The TGA/DSC of the corresponding xerogel sample that was synthesized for comparison is reported in Figure S1A. Xerogels are obtained by slow evaporation of the solvent in air and therefore contain all the organics used during the synthesis, such as the urea and the surfactant. This is clearly shown in the TG curve, that displays an overall weight loss of about 72% under the entire temperature range, with most of the weight loss being below 250°C. The DSC curve suggests that the endothermic decomposition of urea happens between 150 and 230°C and the exothermic combustion of organic residues in the range 240–420°C.

The growth of the ceria nanocrystals within the nano-porous architecture of the silica aerogel was monitored by XRD. In Figure 1B, the XRD patterns of the aerogel samples with a 15% loading of ceria are reported as an example; the patterns refer to the samples obtained after the supercritical drying and after calcination at 450 and 900°C for 1 h in air. In addition to the halo centered at 2θ around 21.5°C typical of the amorphous silica, all patterns display the presence of the reflections due to the fluorite crystalline structure of ceria (Wang et al., 2010). The presence of the peaks in the sample obtained after the supercritical drying indicates that the temperature of 330°C, reached in the autoclave, was high enough to induce the crystallization of the ceria. According to thermal analysis, calcination at 450°C for 1 h in air removes all organics from the surface of the silica aerogel. This step does not affect the crystallinity of the nanocomposite, as no substantial differences are present in the XRD pattern of this sample compared to the same sample before calcination.

**Figure 2:**
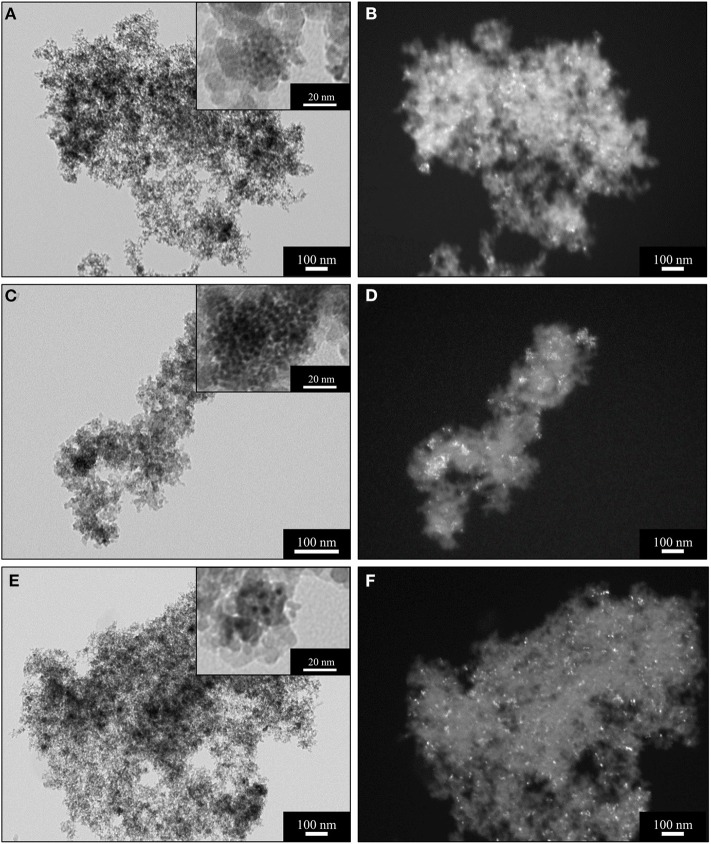
BF (left) and DF (right) TEM images of the sample A_15_CeO_2_ after supercritical drying **(A,B)**; after thermal treatment at 450°C **(C,D)**; after thermal treatment at 900°C **(E,F)**.

To assess the thermal stability of the aerogels, we also performed a thermal treatment at 900°C for 1 h in air. The correspondent XRD pattern in Figure 1B shows more intense and narrower reflections compared to the sample treated at 450°C, suggesting an increase in the mean size of the crystallites. This is confirmed by the average dimensions of the crystallites, which was calculated using the Scherrer equation and is reported in Table 1 for all the samples. In particular, the growth of the ceria nanocrystals induced by the thermal treatments is very limited with the calcination temperature going up to 6 nm in the case of the samples with the highest loading of ceria. In the case of the xerogel sample, an average size of just 2 nm has been determined for the sample treated at 900°C. The XRD patterns of the aerogel samples with 10 and 5% loading of ceria, which are reported in Figures S2B, S3B in Supplementary Information, are very similar to the ones presented in Figure 2 for the aerogel sample with the 15% loading, apart from the intensity of the ceria reflections which is increasing with increasing loading, as expected. The XRD patterns of the xerogel sample, shown in Figure S1B, indicate that the growth of the ceria nanocrystals is very slow, with very broad peaks only appearing after calcination at 900°C. All the XRD patterns measured in this work exclude the formation of undesired crystalline phases, such as cerium silicates (Kepinski et al., 2002). The influence of the length of the thermal treatment was not studied in this study. However, previous studies on nanocomposite aerogels (Loche et al., 2010) indicate that extending the calcination at 750°C up to 20 h has a more limited effect than a calcination at 900°C for 1 h.

**Table 1:** Peak position, FWHM (corrected for an instrumental broadening of 0.18°) and average crystal sizes calculated from the XRD patterns using the Scherrer equation.

**Sample name**	**Peak position [2θ]**	**FWHM [2θ]**	**Crystallite size [nm]**
A_15_CeO_2__NT	47.26	2.49	3
A_15_CeO_2__450	47.47	2.44	4
A_15_CeO_2__900	47.45	1.44	6
A_10_CeO_2__NT	47.26	2.75	3
A_10_CeO_2__450	47.43	2.40	4
A_10_CeO_2__900	47.47	1.58	5
A_5_CeO_2__NT	47.18	2.55	3
A_5_CeO_2__450	47.49	2.41	4
A_5_CeO_2__900	47.47	1.74	5
X_15_CeO_2__450	/	/	/
X_15_CeO_2__900	47.26	4.22	2

*Results refer to all the nanocomposites synthesized using dodecanoic acid with a Ce^3+^/surfactant molar ratio of 1:4. Typical errors on the determination of the average crystal size are ± 1 nm*.

The N_2_-physisorption isotherm of the aerogel sample with a 15% loading of ceria after thermal treatment at 900°C, together with the pore size distribution (inset) calculated from the desorption branch, is reported in Figure 1C. The isotherms corresponding to the aerogel samples with different loading of ceria are reported in Figures S2C, S3C in Supplementary Information where also the isotherm of the xerogel sample is shown, Figure S1C. The N_2_-physisorption data give useful information about the textural features of these samples. The isotherms of the aerogel samples obtained in this work are all very similar to each other and in line with aerogel samples previously synthesized using the same 2-steps sol-gel method (Loche et al., 2010). These can be classified as type IV isotherms with a H3-type hysteresis (Thommes et al., 2015). As expected, in the case of the aerogels, the shape of the isotherm suggests that their porous architecture contains predominantly mesopores, with a macroporous component. In the case of xerogels, Figure S1C, the porous structure is dominated by micropores with a mesoporous component, as suggested by the shape of the isotherm and by the H2(b)-type hysteresis loop typical of these samples.

In Table 2, the values of the surface area (S), calculated by the BET method (Brunauer et al., 1938), the total pore volume (V_P_) and the average pore diameter, calculated by the BJH method (Barrett et al., 1951), are reported for all the samples. In the case of the aerogels, the values of surface area and pore diameter are in agreement with previous studies (Loche et al., 2010). The value of 20 nm for the average pore size confirms that the aerogels are mainly mesoporous, as expected. In the case of the xerogel, a much lower value for the pore diameter has been found, which confirms its microporous nature. It must be highlighted that the 3-dimentional open porous architecture typical of these materials is highly stable under thermal treatments. In particular, in previous reports by our group it has already been shown that aerogel samples synthesized with the same sol-gel method undergo a very limited morphological change upon thermal treatment, retaining high values of surface areas even when they are submitted to high temperature thermal treatments (Loche et al., 2010). This study confirms the high stability of these materials under these extreme conditions. It should be noted that we did not observe a trend in the surface area values with the change in composition. This might be due to a combined effect on the nanocrystals and on the aerogel matrix, whose contribution might go in the opposite direction. In fact, an increase in surface area with increasing loading of the dispersed phase was previously reported (Loche et al., 2012) and attributed to the release of increasing amounts of precursor by products during supercritical drying resulting in a higher amount of micropores.

**Table 2:** Surface area, S, pore volume, V_P_, and pore diameter values for the aerogel and xerogel nanocomposites obtained by N_2_-physisorption.

**Sample name**	**S [m^**2**^ g^**−1**^]**	**V_**P**_ [cm^**3**^ g^**−1**^]**	**Pore diameter [nm]**
A_15_CeO_2__900	359 ± 1	0.86	21
A_10_CeO_2__900	339 ± 1	0.76	21
A_5_CeO_2__900	413 ± 1	0.89	20
X_15_CeO_2__900	394 ± 1	0.28	4

*All these nanocomposites have been synthesized using dodecanoic acid with a Ce^3+^/surfactant molar ratio of 1:4*.

The morphological characterization of the CeO_2_-SiO_2_ aerogel samples was also assessed by TEM observations. In Figure 2, TEM images of the aerogel samples with a 15% loading of ceria as a function of the calcination temperature are shown. The images obtained in bright field mode (BF) (Figures 2A,C,E), display the morphological features typical of the aerogels, such as their low density and open porous architecture. As anticipated by the XRD measurements, the ceria nanoparticles are already present in the sample after the supercritical drying, as it can be assessed by the higher magnification BF image in the inset in Figure 2A, where the ceria nanoparticles appear as darker spots. The close inspection of the TEM images shows that the ceria tend to crystallize in specific regions of the silica matrix, forming clusters of nanoparticles such as the one shown in the inset in Figure 2A. However, the nanoparticles appear separated within these regions, with sizes in the range of 2–5 nm, in agreement with the calculations from the XRD patterns. The distribution of the ceria nanoparticles within the silica is clearer in the dark field (DF) images (Figures 2B,D,F), where the nanocrystals in diffraction conditions appear as bright spots on top of the darker amorphous silica background. The crystalline ceria nanoparticles appear well-distributed within the silica matrix after the supercritical dying process (Figure 2B). After thermal treatment at 450°C (Figures 2C,D), the sample do not show any relevant difference compared with the sample after supercritical drying, with limited growth of the crystallites, as anticipated by the XRD characterization.

The BF TEM image of the sample treated at 900°C in air for 1 h (Figure 2E), demonstrate the high stability of the silica matrix during harsh thermal conditions: the microstructural features typical of the aerogel, such as its porous architecture, are retained and appear identical to the sample after supercritical drying. This is also demonstrated by the high values of surface area calculated from the N_2_-physisorption data and it is also in agreement with previous findings (Loche et al., 2010). Remarkably, also the dispersed phase of ceria nanocrystals shows outstanding stability under thermal treatment of 900°C, as shown in the DF TEM image in Figure 2F, where it can be appreciated that crystal growth or sintering did not occur; this is also in agreement with the XRD measurements, where an average crystallite size of 7 nm was calculated using the Scherrer equation. Moreover, after thermal treatment at 900°C, the ceria nanoparticles appear more homogeneously distributed within the silica matrix compared to the sample treated at 450°C. This might be due to the crystallization of a higher number of nanoparticles and is also in agreement with the XRD pattern shown in Figure 1B.

TEM investigation indicated that the crystallization of ceria takes place within the porous architecture of the silica aerogel, which comprises both meso- and macro pores. It is likely that at relatively low temperatures, such as during the supercritical drying or at 450°C, the nanoparticles tend to crystallize in close proximity to larger pores, where more space and a larger amount of ceria seeds are available, resulting in the aggregates shown in the insets of Figures 2A,C. However, thermal treatment at 900°C enables further crystallization within the smaller pores, yielding overall to a sample with an improved homogeneity in the dispersion of the nanocrystalline phase over the amorphous matrix.

**Figure 3:**
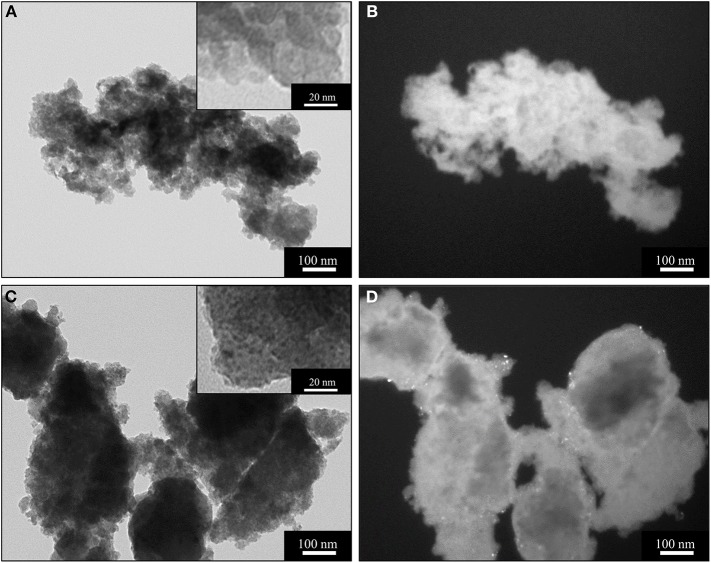
BF (left) and DF (right) TEM images of the sample X_15_CeO_2_ thermally treated at 450°C **(A,B)** and 900°C **(C,D)**.

TEM characterization of the xerogel (Figure 3), also provides some useful insights into the crystallization of the ceria nanophase within the silica matrix. This sample has been prepared using the same synthetic procedure and the same concentration of cerium precursor used for the aerogel sample. However, the different drying process—i.e., slow evaporation—causes the collapse of the original porous structure of the gel, yielding a mainly microporous silica matrix with the absence of macropores. This is confirmed by the N_2_-physisorption characterization shown in Figure S1C. In Figure 3, BF and DF TEM images of the xerogel sample treated at 450°C (Figures 3A,B) and 900°C (Figures 3C,D) are reported. In the TEM image in Figure 3A, the typical morphology of a silica xerogel matrix can be observed: as expected, the silica matrix appears much denser than the aerogel. Both the inset in Figure 3A and the DF image in Figure 3B show the absence of crystalline ceria nanoparticles. This is in agreement with the XRD pattern of this sample, reported in Figure S1B. After the thermal treatment at 900°C, the dispersed ceria nanoparticles appear clearly visible in both the BF (dark spots in the inset) and DF images (bright spots) (Figures 3C,D). In the DF image, the bright spots are visible only in the edges of the sample due to the higher density of the silica matrix compared to the aerogel. The nanoparticles appear homogeneously distributed with the absence of the aggregates that occurred in the aerogel samples: this is likely due to the nanoparticles being forced to crystallize within the micropores of the xerogel. The TEM observations in Figures 3C,D are in agreement with the XRD pattern of the sample (Figure S1B), where the peaks of the ceria only appear as consequence of the thermal treatment at 900°C. The calculation using the Scherrer equation indicates an average crystallite size of 2 nm, Table 1, which is consistent with the nanoparticles crystallizing inside the micropores.

The presence of the carboxylic fatty acid turned out to play a key role in the successful synthesis of the nanocomposites shown in this study. The synthesis of the same CeO_2_-SiO_2_ aerogel composite without the use of a surfactant was also attempted. The XRD pattern of the sample obtained after the supercritical drying showed the presence of a crystalline phase corresponding to cerium carbonate hydroxide, CeCO_3_OH (Figure S4) (Akinc and Sordelet, 1987). TEM characterization showed the presence of submicrometer spheres, with diameters spanning from 150 to 500 nm randomly distributed within the porous texture of the silica aerogel (Figure S5). However, as shown in this study, the use of a carboxylic acid such as dodecanoic acid (or hexanoic acid) enabled the preparation of remarkably thermally stable CeO_2_-SiO_2_ aerogel nanocomposites with a high degree of homogeneity in terms of size and distribution of the ceria phase within the silica matrix. The need of the surfactant to control the growth of the ceria nanocrystals is likely due to the fact that crystal nucleation and growth starts while the supercritical drying is performed, in contrast to other nanocomposites containing less reactive transition metals where nucleation and growth is achieved during the calcination steps. It should be noted that the fatty acid is able to direct the synthesis toward a homogeneous distribution of the ceria nanoparticles in the nanocomposite and to avoid the formation of very large ceria crystals, regardless of the length of the alkyl chain.

Finally, the influence of the concentration of the dodecanoic acid was explored by synthesizing samples with molar ratios of Ce^3+^/dodecanoic acid 1:1 and 1:8, which were compared to standard conditions of Ce^3+^/dodecanoic acid 1:4, and of the length of the alkyl chain of the surfactant by synthesizing an aerogel sample using hexanoic acid (1:4 Ce^3+^/hexanoic acid) in place of the dodecanoic acid. In all these cases we obtained nanocomposites with textural and microstructural features similar to the ones obtained using dodecanoic acid with a 1:4 ratio. The TGA/DSC, XRD and N_2_-physisorption characterization of these samples are shown in Figures S6–S8, together with the TEM images of these samples after thermal treatment at 900°C, reported in Figure S9. The crystallization of the nanocrystalline ceria within the aerogel matrix appears to be independent on the concentration of the carboxylic acid used during the synthesis and on the length of its alkyl chain. Overall, these data indicate that the role of the surfactant is mainly due to the effect of the carboxylic functionality, which is able to coordinate and mediate ceria crystallization without altering silica aerogel formation.

## Conclusions

In this work, we showed the synthesis and characterization of novel CeO_2_-SiO_2_ aerogel nanocomposites where ceria nanoparticles were crystallized within the nanoporous architecture of the silica aerogel. These nanocomposites were obtained via a 2-step sol-gel protocol making use of carboxylic acids as surfactants allowing an extensive control over the loading, size and distribution of the dispersed ceria nanoparticles. Our results showed that the crystallization of the ceria nanoparticles is influenced by the textural features of the silica and by the presence of the carboxylic acids. Moreover, we demonstrated the exemplary stability of these nanocomposites under thermal treatments at temperatures as high as 900°C. These nanocomposites might find application in the field of heterogeneous gas-phase catalysis for industrially relevant catalytic processes such as the CO oxidation and the water gas shift reaction.

## Data Availability Statement

The datasets generated for this study are available on request to the corresponding author.

## Author Contributions

FC and DL synthesized the samples and carried out the XRD characterization. FC carried out the TGA and N_2_-physisorption characterization and wrote the manuscript that was then revised with contributions from all the authors. DL and MC carried out the TEM characterization. AC supervised the work.

### Conflict of Interest

The authors declare that the research was conducted in the absence of any commercial or financial relationships that could be construed as a potential conflict of interest.
