# Supplementary material for: Growing CeO2 Nanoparticles Within the Nano-Porous Architecture of the SiO2 Aerogel
Source: Front Chem. 2020 Feb 7;8:57. doi: 10.3389/fchem.2020.00057 (PMC7018665; doi:10.3389/fchem.2020.00057)
Supplement: Supplementary file 1 [file Table_1.DOCX]

**Figure S1.** (A) TGA/DSC measurement of the xerogel sample X_15_CeO_2__NT; TG (bold line), DSC (dashed line); (B) XRD patterns of the same sample after thermal treatments at 450 °C and 900 °C; (C) N_2_-physisorption isotherm of the same sample after thermal treatment at 900 °C (adsorption branch: bold line; desorption branch: dashed line) and pore size distribution calculated from the desorption branch (inset).

**Figure S2.** (A) TGA/DSC measurement of the aerogel sample A_10_CeO_2__NT; TG (bold line), DSC (dashed line); (B) XRD patterns of the same sample not treated and after thermal treatments at 450 °C and 900 °C; (C) N_2_-physisorption isotherm of the same sample after thermal treatment at 900 °C (adsorption branch: bold line; desorption branch: dashed line) and pore size distribution calculated from the desorption branch (inset).

**Figure S3.** (A) TGA/DSC measurement of the aerogel sample A_5_CeO_2__NT; TG (bold line), DSC (dashed line); (B) XRD patterns of the same sample not treated and after thermal treatments at 450 °C and 900 °C; (C) N_2_-physisorption isotherm of the same sample after thermal treatment at 900 °C (adsorption branch: bold line; desorption branch: dashed line) and pore size distribution calculated from the desorption branch (inset).

**Figure S4.** XRD pattern of the aerogel sample, synthetized without the use of the capping agent, after supercritical drying. All the reflections correspond to a cerium carbonate hydroxide, CeCO_3_OH crystalline phase. The asterisk indicates a reflection corresponding to the presence of ceria.

**Figure S5.** BF TEM images of the aerogel composite, synthetized without the use of the capping agent, after supercritical drying.

**Figure S6.** (A) TGA/DSC measurement of the aerogel sample A_5_CeO_2__NT with dodecanoic acid/Ce^3+^ molar ratio 1:1; TG (bold line), DSC (dashed line); (B) XRD patterns of the same sample not treated and after thermal treatments at 450 °C and 900 °C; (C) N_2_-physisorption isotherm of the same sample after thermal treatment at 900 °C (adsorption branch: bold line; desorption branch: dashed line) and pore size distribution calculated from the desorption branch (inset).

**Figure S7.** (A) TGA/DSC measurement of the aerogel sample A_5_CeO_2__NT with dodecanoic acid/Ce^3+^ molar ratio 1:8; TG (bold line), DSC (dashed line); (B) XRD patterns of the same sample not treated and after thermal treatments at 450 °C and 900 °C; (C) N_2_-physisorption isotherm of the same sample after thermal treatment at 900 °C (adsorption branch: bold line; desorption branch: dashed line) and pore size distribution calculated from the desorption branch (inset).

**Figure S8.** (A) TGA/DSC measurement of the aerogel sample A_10_CeO_2__NT synthesized using hexanoic acid (1:4 molar ratio); TG (bold line), DSC (dashed line); (B) XRD patterns of the same sample not treated and after thermal treatments at 450 °C and 900 °C; (C) N_2_-physisorption isotherm of the same sample after thermal treatment at 900 °C (adsorption branch: bold line; desorption branch: dashed line) and pore size distribution calculated from the desorption branch (inset).

Figure S9 TEM images of the aerogel samples synthesized using dodecanoic acid with a molar ratio of 1:1 (A, B), 1:8 (C, D) and using hexanoic acid with a 1:4 molar ratio (E, F) after thermal treatment at 900 °C. BF in the left, DF in the right. Higher magnification images in the insets.
